# Surveillance systems evaluation: a systematic review of the existing approaches

**DOI:** 10.1186/s12889-015-1791-5

**Published:** 2015-05-01

**Authors:** Clementine Calba, Flavie L Goutard, Linda Hoinville, Pascal Hendrikx, Ann Lindberg, Claude Saegerman, Marisa Peyre

**Affiliations:** Département ES, UPR AGIRs, Bureau 208, Bâtiment E TA C22/E, Centre de coopération internationale en recherche agronomique pour le développement (CIRAD), Campus international de Baillarguet, Montpellier Cedex 5, 34398 France; Animal Health and Veterinary Laboratories Agency (AHVLA), Weybridge, New Haw, Addlestone KT15 3NB UK; French Agency for Food, Environmental and Occupational Health Safety (ANSES), 31 avenue Tony Garnier, 69394 Lyon Cedex 07, France; National Veterinary Institute (SVA), SE-751 89 Uppsala, Sweden; Research Unit of Epidemiology and Risk Analysis applied to Veterinary Sciences (UREAR-ULg), Fundamental and Applied Research for Animal and Health, Faculty of Veterinary Medicine, University of Liege, Boulevard de Colonster 20, B42, B-4000 Liege, Belgium; CIRAD, UPR AGIRS, Bureau 208, Bâtiment E, TA C22/E, Campus international de Baillarguet, 34398 Montpellier Cedex 5, France

**Keywords:** Surveillance, Evaluation approaches, Health

## Abstract

**Background:**

Regular and relevant evaluations of surveillance systems are essential to improve their performance and cost-effectiveness. With this in mind several organizations have developed evaluation approaches to facilitate the design and implementation of these evaluations.

**Methods:**

In order to identify and to compare the advantages and limitations of these approaches, we implemented a systematic review using the PRISMA guidelines (Preferred Reporting Items for Systematic Reviews and Meta-Analyses).

**Results:**

After applying exclusion criteria and identifying other additional documents via citations, 15 documents were retained. These were analysed to assess the field (public or animal health) and the type of surveillance systems targeted; the development process; the objectives; the evaluation process and its outputs; and the attributes covered. Most of the approaches identified were general and provided broad recommendations for evaluation. Several common steps in the evaluation process were identified: *(i)* defining the surveillance system under evaluation, *(ii)* designing the evaluation process, *(iii)* implementing the evaluation, and *(iv)* drawing conclusions and recommendations.

**Conclusions:**

A lack of information regarding the identification and selection of methods and tools to assess the evaluation attributes was highlighted; as well as a lack of consideration of economic attributes and sociological aspects.

## Background

The concepts underpinning surveillance and the number of different surveillance systems in use have expanded rapidly in recent years [1]. These systems have been developed in various fields, either public health (PH), animal health (AH), environmental health (EH), or more recently, combining these sectors in a one health (OH) approach [2].

Although the need for effective surveillance systems has long been recognized, there is increasing international pressure to improve the effectiveness of those systems even further [3]. The capacity of surveillance systems to accurately describe patterns of diseases is of public health importance. Therefore, regular and relevant evaluations of these systems are critical in order to improve their performance and efficiency [4]. Depending on epidemiological, sociological and economic factors, disease surveillance systems can be complex, meaning that multiple attributes are required to assess their performance and many different methods and tools are needed to evaluate them.

Several organizations or institutions have developed their own approaches for conducting evaluations of surveillance systems, and for providing relevant recommendations. These approaches path the way for developing evaluation strategies, to help evaluators to generate data in order to address specific evaluation question(s) required for management and decision-making [5]. Nonetheless, Klaucke highlighted that ‘each evaluation should be individually tailored’, due to the fact that surveillance systems vary widely in their methods, scope, and objectives [6]. This highlights the need for these evaluation approaches to be flexible enough to allow for these variations in surveillance systems.

The purposes of this review were therefore to identify and analyse the existing health surveillance systems evaluation approaches in order to allow end users (i.e. decision makers in health surveillance programs at all administrative levels of implementation) to select the most appropriate approach based on their objective(s) and also to inform the development of an evaluation framework within the RISKSUR project ^a^ (Risk-based animal health surveillance systems). This review follows up on a review performed recently by Drewe and co-workers [3] which focused on the technical aspects of the evaluation process (i.e. which attributes to assess and which assessment methods to use), by taking a broader approach to examine the approaches developed to conduct these evaluations. The specific objectives of this study were to assess the advantages and limitations of these approaches; and to identify the existing gaps in order to assess the potential needs for improvement in the evaluation guidance process and thereby inform the design of a comprehensive evaluation framework.

## Methods

### Literature sources and search strategy

A systematic literature search was conducted according to the PRISMA requirements (Preferred Reporting Items for Systematic Reviews and Meta-Analyses) [7], using CAB abstract (Commonwealth Agricultural Bureau), Web of Science, Medline, Google Scholar, and Scopus to identify articles. The literature search focused on papers published between 1992 and January 2013. It was restricted to the English language, and to articles with available abstracts. Four domains were included in the search, with several keywords for each: surveillance (“surveillance or report* or monitor*”), evaluation (“evaluat* or assess* or analys*”), framework (“framework or guideline or method* or tool”), and health (“health or bioterrorism or public security”).

Four search algorithms using the corresponding Medical Subject Headings (MeSH) key words were used, targeting the same domains as the previous search:

- [“health information system” OR “health surveillance” OR “health information network”] + “evaluation guidelines” + [methods OR tools]
- [“health information system” OR “health surveillance” OR “health information network”] + “evaluation framework” + [methods OR tools]
- [“health information system” OR “health surveillance” OR “health information network”] + “assessment guidelines” + [methods OR tools]
- [“health information system” OR “health surveillance” OR “health information network”] + “assessment framework” + [methods OR tools]

Some exclusion criteria were directly used during this second search process: “surgical procedures”, “drug treatment”, “risk management”, “risk analysis”, “cancer”, “clinical trial”, and “risk assessment”.

Additionally, six documents were identified from the references of included articles and were subsequently added to the systematic review.

### Study selection and data extraction

The literature retrieval process was done through two screening phases. The first screening phase was applied to the titles and abstracts; the second phase was applied to the full texts. For each phase, nine exclusion criteria were applied: articles not stating at least one of the following terms (public health, animal health/disease, environmental health, bioterrorism, public security, performance indicators) *(i)*; articles describing evaluations of test performance *(ii)* or success rate of surgical procedures *(iii)* or drug treatment *(iv)*; and results of a surveillance system rather than the performance of the system itself *(v)*; articles related to the evaluation of surveillance tools rather than evaluation of the system *(vi)*, articles describing the importance of the evaluation rather than the evaluation process *(vii)*, articles not related to the evaluation of surveillance *(viii)*, and articles describing results from an evaluation rather than describing the method *(ix)*.

From the articles finally selected, the following data were extracted: the surveillance field (human or animal health), the category of surveillance system considered and the type of evaluation proposed; the evaluation approach development process; the evaluation objectives; the evaluation process; the assessment process; and practical applications (if any). A comparative analysis of completeness and practicality of the different evaluation approaches was performed. In this way, all practical elements for evaluation were extracted from the references and a complete list was designed.

### Classification of the approaches

A variety of terms were used to describe the existing approaches and it was not clear why authors had selected these. Therefore, we have used the following definitions for these terms in this review:

- A framework is considered to be skeletal support used as the basis for something being constructed; it is an organization of concepts that provides a focus for inquiry [8,9].
- A guideline can be defined as a document to be followed in the performance of certain tasks; this provides recommendations (a set of standards or criteria) for the steps that should be used to achieve a desired goal [10,11].
- A method provides information about how to accomplish an end; it is a regular and systematic way of accomplishing something [12].
- A tool can be defined as a process with a specific purpose; it is used as a mean of performing an operation or achieving an end [13,14].

In other words, frameworks would help users to define what to take into consideration in the evaluation process; guidelines would inform the different steps needed to conduct the evaluation; methods would detail how to implement the evaluation (what to assess and how); and tools would not only provide a methodology but also include practical elements to be used to conduct the evaluation (e.g. spreadsheets, questionnaires).

## Results

**Figure 1:**
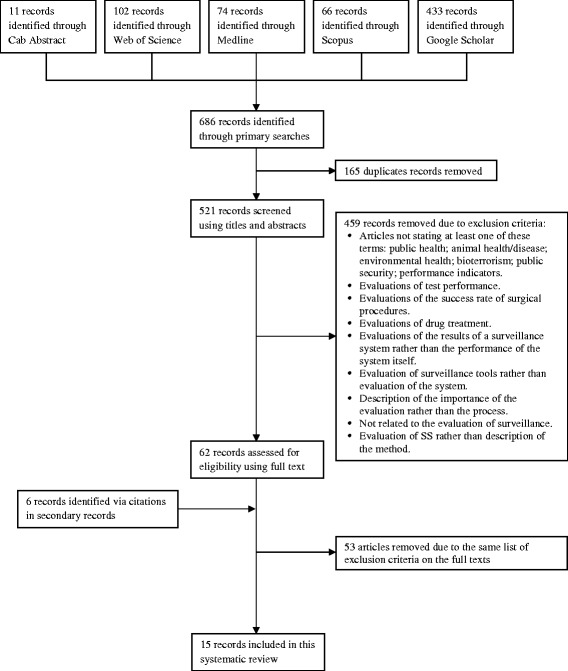
PRISMA flow chart diagram of studies selection process to include in the systematic review.

The literature search identified a total of 521 records (Figure 1). Three were not available and have been excluded [15-17]. The remaining articles were screened and a total of 15 articles remained (Figure 1).

### Field of application and category of evaluation approaches

Among the identified approaches, ten originated from the public health surveillance field [5,18-26]; three from animal health surveillance [27-29]; one from environmental health surveillance [30]; and one both from animal and public health surveillance [31] (Table 1).

**Table 1:** **Category, surveillance field and objective(s) of the approaches used for the evaluation of surveillance systems**

**References**	**Approach category**		**Surveillance field**	**Main objective**	**Objective(s) of the evaluation as stated in the document**	**Case study application**
	**Author’s definition**	Reviewed definition*				
[5]	Framework	Framework Guidelines Method	PH^a^	Evaluate performance and effectiveness	To assess the quality of the information provided; the effectiveness in supporting the objective(s), in supporting informed decision-making; and the efficiency of SS	-
[18]	Tool	Method Tool	PH^a^	Design efficient surveillance systems	Help plan, organize, implement SS	Not described
[19]	Tool	Guidelines Method Tool	PH^a^	Design efficient surveillance systems	To establish a baseline and to monitor progress	-
[20]	Guidelines	Framework Guidelines Method	PH^a^	Evaluate performance and effectiveness	To establish and maintain effective and efficient surveillance and response systems	-
[21]	Framework	Guidelines	PH^a^	Evaluate performance and effectiveness	To assess existing SS and identify areas which can be improved	-
[22]	Framework	Framework	PH^a^	Evaluate performance and effectiveness	To evaluate whether SS attain their objectives, and to provide information for further development and improvement	Military surveillance systems for early detection of outbreaks on duty areas
[23]	Framework	Frameworl	PH^a^	Evaluate performance and effectiveness	To provide objective, valid and reliable information for the decisions on which surveillance activities and functions should be continued	-
[24]	Framework	Framework Guidelines	PH^a^	Evaluate performance and effectiveness	To establish the relative value of different approaches and to provide information needed to improve their efficacy	-
[25]	Tool	Framework Guidelines	PH^a^	Evaluate performance and effectiveness	To assess whether the surveillance method appropriately addresses the disease/health issues; whether the technical performance is adequate	-
[26]	Guidelines	Framework Guidelines	PH^a^	Evaluate performance and effectiveness	To define how well the system operates to meet its objective(s) and purpose	-
[27]	Tool	Method Tool	AH^b^	Evaluate performance and effectiveness	To propose recommendation for improvement of SS	Implemented in France: surveillance network for antimicrobial resistance in pathogenic bacteria from animal origin (also mentioned but not described: early detection of FMD; case detection of rabies in bats; poultry disease surveillance network and salmonella laboratory surveillance network)
[28]	Framework	Framework Guidelines Method	AH^b^	Evaluate performance and effectiveness	Support the detection of disparities in surveillance and support decisions on refining SS design	Implemented in UK: demonstration of freedom from Brucella melitensis; early detection of CSF and case detection of Tb.
[29,32]	Method	Guidelines Method	AH^b^	Evaluate performance and effectiveness	To contribute to the improvement of the management of epidemiological animal health SS	Implemented in France: evolution of mycoplasmosis and salmonellosis rates in poultry (RENESA network); and the FMD surveillance network in cattle
[30]	Framework	Framework	EH^c^	Evaluate performance and effectiveness	Make evidence-based decisions regarding the future selection, development and use of data	Environmental public health surveillance programs
[31]	Method	Guidelines Method	PH^a^ & AH^b^	Evaluate the completeness of the surveillance systems in terms of core components	Evaluate the completeness and coherence of the concepts underlying a health surveillance program	National Integrated Enteric Pathogen Surveillance Program, Canada

a: Public Health; b: Animal Health; c: Environmental Health; SS: Surveillance System. FMD: Foot and Mouth Disease *According to the information provided in the publication.

Seven approaches were defined by their authors as frameworks [5,21-24,28,30]; two as guidelines [20,26]; two as methods [29,31]; and four as tools [18,19,25,27] (Table 1). However according to the reviewed definitions provided above, most of the approaches (13/15) could be defined either as frameworks or guidelines as they would provide a general or structured roadmap for the evaluation process [5,19-26,28-31] (Table 1). Eight approaches provided systematic information about how the evaluation should be carried out and could therefore be defined as methods [5,18-20,27-29,31], but only three approaches provided practical tools to implement the evaluation (two in PH [18,19] and one in AH [27]) (Table 1).

### Approach development processes and case study applications

The development process was clearly described in four out of the 15 approaches [27-30] (Table 1). Three approaches were designed through expert opinion [27-29]. The SERVAL framework (Surveillance evaluation framework) [28] was developed by 16 experts in surveillance, and reviewed by 14 others. The Critical Control Point (CCP) method [29] was derived from the Hazard Analysis Critical Control Point method (HACCP), and submitted to a panel of experts using a Delphi consultation method. The OASIS tool (Outil d’analyse des systèmes de surveillance) [27] was designed through the combination of three assessment methods (Surveillance Network Assessment Tool, CCP, and the Centre for Disease Control and prevention (CDC) and the World Health Organisation (WHO) guidelines), and was submitted to an expert opinion elicitation process. The framework on environmental public health surveillance programs [30] was developed based on results from a stepwise review of environmental surveillance and monitoring systems data, in order to identify indicators and examine criteria used in environmental health. The framework for evaluating military surveillance systems for early detection [22] was based on the CDC framework for evaluating public health surveillance systems for early detection of outbreaks [24]. However the CDC framework development process was not described in the publication.

Two approaches were developed based on case studies, which are not described in the articles [22,30]; one was specifically developed for European Union surveillance systems [23], and four have been supported by case studies which are directly described in the corresponding publication [27-29,31] (Table 1). The SERVAL framework [28] was tested on three British surveillance systems, targeting different surveillance objectives: demonstration of freedom of *Brucella melitensis* in sheep and goats by serological surveillance; early detection of classical swine fever in pigs (exotic disease); and surveillance of endemic tuberculosis in cattle. The conceptual evaluation of veterinary and public health surveillance programs method [31] was applied to the evaluation of the National Integrated Enteric Pathogen Surveillance Program in Canada (C-EnterNet; http://www.phac-aspc.gc.ca/c-enternet/index-eng.php). The OASIS evaluation tool [27] has been applied to the evaluation of five surveillance systems, but only the evaluation of the French surveillance network for antimicrobial resistance in pathogenic bacteria from animal origin (RESAPATH) was described in the article [27]. The CCP method developed by Dufour [29] was tested on two surveillance systems described in the “Epidemiological surveillance in animal health” book [32]. These case studies targeted the French RENESA network (Evolution of mycoplasmosis and salmonellosis rates in poultry); and the French Foot and Mouth Disease (FMD) surveillance network in cattle.

### Objectives of the evaluation and description of the evaluation process

According to the area and to the type of surveillance, three main objectives were identified (Table 1): evaluate surveillance systems performance and effectiveness (for 12 approaches [5,20-30]), design efficient surveillance systems (2 approaches [18,19]), and evaluate the completeness of the surveillance systems in terms of core components (one approach [31]).

Fourteen out of the 15 approaches provided an evaluation process structured around 3 to 6 steps [5,19-31] (Table 2), highlighting four common stages in the evaluation process: *(i)* defining the surveillance system under evaluation, *(ii)* designing the evaluation process, *(iii)* implementing the evaluation, and *(iv)* drawing conclusions and recommendations.

**Table 2:** **Steps of the evaluation process provided by the identified evaluation approaches; along with absence or presence of the different practical element retrieved from the analysis**

**References**	**Organisation**	**Steps**	**Practical evaluation elements**	
			**Presence**	**Absence**
[5]	Structured roadmap	Context of the surveillance system	- List of evaluation attributes (13)	- No case study presentation
		Evaluation questions		- Lack of visual representation of the results
		Process for data collection and management		- Lack of information about evaluator(s)
		Findings	- Definitions of evaluation attributes	- Lack of methods and tools for the assessment (only general questions)
		Evaluation report		- Lack of attributes’ selection matrix
		Following up		
[18]	Structured roadmap - Worksheets (checklist)	-	- Methods and tools for the assessment: questionnaire and worksheets	- No case study presentation
				- Lack of information about evaluator(s)
			- Visual representation of the results: bar and radar charts	- Lack of evaluation attributes
				- Lack of definitions of evaluation attributes
				- Lack of attributes’ selection matrix
[19]	Structured roadmap - Application guide	Resources assessment		- No case study presentation
		Indicators		- Lack of information about evaluator(s)
		Data sources assessment		
		Data management assessment	- Methods and tools for the assessment: scoring guide	- Lack of evaluation attributes
		Data quality assessment	- Visual representation of the results (graphs)	- Lack of definitions of evaluation attributes
		Information dissemination and use		- Lack of attributes’ selection matrix
[20]	Structured roadmap	Plan to evaluation	- List of evaluation attributes (10)	- No case study presentation
				- Lack of visual representation of the results
		Prepare to evaluate		- Lack of information about evaluator(s)
		Conduct the evaluation	Definitions of evaluation attributes	- Lack of methods and tools for the assessment (only general questions)
		Dissemination and use of the results		- Lack of attributes’ selection matrix
[21]	Structured roadmap	Preparation for the evaluation	- Type/knowledge of evaluator(s): Ministry of Health (national, provincial or district levels)	- No case study presentation
		Documentation and evaluation of the surveillance system	- List of evaluation attributes (8)	- Lack of visual representation of the results
		Evaluation of the capacity of the surveillance system	- Definitions of evaluation attributes	- Lack of methods and tools for the assessment (general questions)
		Outcome of the evaluation		- Lack of attributes’ selection matrix
[22]	General roadmap	Initial evaluation	- List of evaluation attributes (16)	- No case study presentation
				- Lack of visual representation of the results
		Intermediate evaluation	-Definitions of evaluation attributes	- Lack of information about evaluator(s)
		Final evaluation		- Lack of methods and tools for the assessment
				- Lack of attributes’ selection matrix
[23]	General roadmap	Usefulness of the activities and outputs	- Type/knowledge of evaluator(s): three to four evaluators (5 years of expertise in surveillance on communicable diseases for the team leader, plus a laboratory expert and an expert in epidemiology)	- No case study presentation
				- Lack of visual representation of the results
				- Lack of definitions of evaluation attributes
		Technical performance		- Lack of methods and tools for the assessment
		Fulfilment of contract objectives	- List of evaluation attributes (7)	- Lack of attributes’ selection matrix
[24]	General roadmap	System description	- List of evaluation attributes (9)	- No case study presentation
				- Lack of visual representation of the results
		Outbreak detection		- Lack of information about evaluator(s)
		System experience	- Definitions of evaluation attributes	- Lack of methods and tools for the assessment (general questions)
		Conclusions and recommendations		- Lack of attributes’ selection matrix
[25]	Structured roadmap - Questionnaire	Usefulness of the operation	- Type/knowledge of evaluator(s): experts in international surveillance on communicable diseases	- No case study presentation
		Quality of the outputs		- Lack of visual representation of the results
		Development of the national surveillance system		- Lack of definitions of evaluation attributes
		Technical performance	- List of evaluation attributes (6)	- Lack of methods and tools for the assessment (general questions)
		Structure and management		- Lack of attributes’ selection matrix
[26]	General roadmap	Engage the stakeholders	- List of evaluation attributes (10)	- No case study presentation
		Describe the surveillance system		- Lack of visual representation of the results
		Evaluation design		- Lack of information about evaluator(s)
		Performance of the surveillance system	- Definitions of evaluation attributes	- Lack of methods and tools for the assessment (general questions)
		Conclusions and recommendations		- Lack of attributes’ selection matrix
		Findings and lessons learned		
[27]	Structured roadmap - Questionnaire - Scoring guide - Worksheets	Design the evaluation	- Case study presentation (c.f. Table 1)	- Lack of definitions of evaluation attributes
			- Visual representation of the results through diagram representations (pie charts, histogram, radar chart)	
		Implement the evaluation	- Type/knowledge of evaluator(s): requires little knowledge and experience related to surveillance	
		Finalisation	- List of evaluation attributes (10) and performance indicators	- Lack of attributes’ selection matrix
			- Methods and tools for the assessment: questionnaire, scoring guide and worksheets	
[28]	Structured roadmap - Application guide	Scope of evaluation	- Case study application (c.f. Table 1) (Table 1)	- Lack of methods and tools for the assessment (only references provided)
		Surveillance system characteristics	- Visual representation of the results through colour-coding (green, orange, red)	
		Design the evaluation	- Type/knowledge of evaluator(s): “Anyone familiar with epidemiological concepts and with a reasonable knowledge of the disease under surveillance”	
		Conduct the evaluation		
		Report	- List of evaluation attributes (22)	
			- Definitions of evaluation attributes	
			- Attributes’ selection matrix	
[29,32]	Structured roadmap - Questionnaire - Scoring guide	Description of the surveillance system	Case study presentation (c.f. Table 1)	- Lack of visual representation of the results
		Identification of the priority objectives		- Lack of information about evaluator(s)
				-Lack of evaluation attributes
		Building of dashboard and indicators	Provides performance indicators	- Lack of definitions of evaluation attributes
		Implementation and follow-up		- Lack of methods and tools for the assessment
		Updates and audit		- Lack of attributes’ selection matrix
[30]	General roadmap	Priority setting	- Provides performance indicators	- No case study presentation
				- Lack of visual representation of the results
				- Lack of information about evaluator(s)
		Scientific basis and relevance		- Lack of evaluation attributes
		Analytic soundness and feasibility		- Lack of definitions of evaluation attributes
		Interpretation and utility		- Lack of methods and tools for the assessment
				- Lack of attributes’ selection matrix
[31]	General roadmap	Text analysis	- Case study presentation (c.f. Table 1)	- Lack of visual representation of the results
				- Lack of information about evaluator(s)
		Program conceptual model		
				- Lack of evaluation attributes
		Comparison Validation		- Lack of definitions of evaluation attributes
				- Lack of methods and tools for the assessment
				- Lack of attributes’ selection matrix

### Description of the assessment process: evaluation attributes

**Figure 2:**
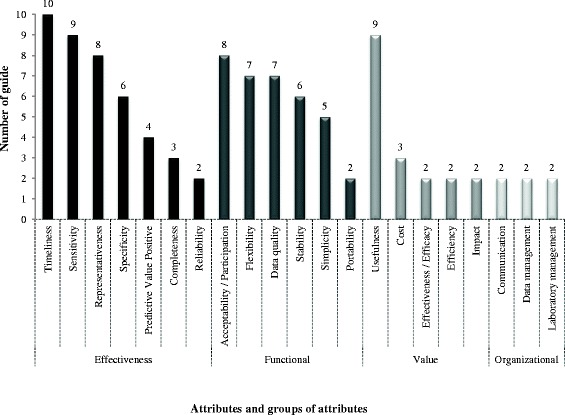
Number of evaluation approaches which take into consideration each evaluation attribute identified in this review.

A total of 49 distinct evaluation attributes were identified through this systematic review. Attributes which were considered only in one evaluation approach have been removed from the analysis for more clarity. The number of approaches taking into consideration each attribute is presented in Figure 2. The attributes could be grouped into 4 different categories linked to the aspect of the surveillance systems they evaluate: effectiveness, functional, value, and organizational attributes [33].

The evaluation approaches most frequently focused on attributes related to the effectiveness of the system (Figure 2), especially timeliness which was included in all the identified approaches [5,20-28], and sensitivity in 9/10 [5,20-22,24-28]. Regarding the functional attributes, the evaluation approaches mainly recommended the assessment of acceptability (8/10) [5,20-22,24,26-28], flexibility (7/10) [5,20,21,24,26-28], stability (6/10) [5,22,24,26-28] and simplicity (5/10) [5,20,21,26,27]. Other attributes such as usefulness (9/10) [5,20-27], representativeness (8/10) [5,20,21,24-28] and data quality (7/10) [5,22-27] were also included in more than half of the approaches. Attributes aimed at assessing the value of surveillance system were not often considered, especially economic attributes: assessment of the cost was recommended in only 3/10 approaches [22,24,28]; impact, effectiveness/efficacy and efficiency in only 2/10 [5,22,28].

Regarding the assessment process of these attributes, guidance was only provided by giving examples of questions to ask key stakeholders in six approaches (Table 2) [5,20,21,24-26]. These questions were mostly general, and it was not always specified who should be interviewed to collect relevant information. One approach included references to published implementation of methods or tools for the assessment of specific attributes, which could be used as example or basis but no guidance was included about how to select the most appropriate method (Table 2) [28].

Ten out of the 21 attributes included in the approaches illustrated in Figure 2 were the ones recommended in the CDC guidelines [24]. This correlates with previous findings from Drewe and co-workers [3] who highlighted that almost a quarter of identified published studies that have evaluated surveillance systems have used attributes recommended by the CDC guidelines [24].

### Comparison between approaches

For each identified evaluation approach, the practical elements proposed to perform the evaluation were listed (e.g. list of evaluation attributes, case study presentation). A combined list of these elements and their usefulness in the evaluation process are presented in Table 3. The presence or absence of these practical elements in each evaluation approach considered in this review was analysed. This allowed the comparative analysis of the existing approaches according to their completeness and practicality (Table 3):

1. The lack of a case study application. Four approaches were applied to case studies [27-29,31], which ease further application by users.
2. The failure to provide a graphical representation of the outputs. Graphical outputs (e.g. pie charts, histograms) were produced by four approaches [18,19,27,28], which ease the reporting, communication and further analysis of the evaluation results.
3. The absence of recommendations on the type of evaluator and the expertise they require. Five approaches gave information about the evaluator requirements (e.g. expertise in surveillance on communicable diseases, senior laboratory expert) [21,23,25,27,28], which helps to assess the feasibility and ensure the quality of the evaluation.
4. Insufficient practical information about which evaluation attributes to assess (e.g. sensitivity, acceptability). Ten approaches provided a list of attributes [5,20-28] and the attributes were defined in 7 of these [5,20-22,24,26,28]. However only one approach [28] provided information on how to prioritize these attributes according to the surveillance context and objective by the mean of a matrix grid.
5. An absence of information about how to assess the evaluation attributes. Even though ten approaches provided information on which attributes to assess, only the OASIS tool provided detailed methods and a ready to use tool to perform this assessment [27]. Six approaches suggested ways on how to handle the assessment phase, by providing general questions related to the assessment of each attribute (e.g. is the time interval appropriate for the health intervention?) [5,20,21,24-26]; and one provided references to relevant publications related to practical evaluation of surveillance systems and to existing methods and tools [28]. Moreover, none of the approaches provided information about how to interpret the attributes assessments.

**Table 3:** **Practical aspects identified in a review of evaluation approaches for health surveillance systems, and their role in the evaluation process**

**Practical elements**	**Usefulness**
List of evaluation attributes to be assessed	Design the evaluation
Definitions of the evaluation attributes to be assessed	Design the evaluation
Case study presentation	Ease of applicability
Visual representation of the results	Ease of communication
Information about evaluator(s) (e.g. required expertise level)	Design the evaluation
List of methods and tools to assess the evaluation attributes targeted	Design the evaluation
	Ease of applicability
Guide for the selection of relevant evaluation attributes	Design the evaluation
	Ease of applicability

## Discussion

Although the evaluation objectives of the various approaches varied according to the field of application and to the type of approach, four common steps in the evaluation process were identified: *(i)* description of the context, *(ii)* description of the evaluation process, *(iii)* implementation, and *(iv)* recommendations. Three evaluation approaches focused on the evaluation of the structure of the system [18,19,31] but the majority also included an evaluation of the quality of the data generated and the system’s performance. Those approaches also considered implicitly the structure of the system which has to be described in order to understand the surveillance process, to select relevant attributes to be assessed and to provide relevant recommendations.

One of the main limitations of the existing approaches was the level of detail provided to the evaluators in order to practically implement the evaluation. Most of the identified approaches provided generic recommendations for evaluations (i.e. framework and guidelines) with more or less level of detail on the different steps to implement. Only three included methods and tools for the implementation of the evaluation (i.e. ready-to-use questionnaires and/or scoring guides) [18,19,27], of which only one related to AH [27]. This highlights the need for practical tool development in this field. The requirement for flexibility to account for variations in the surveillance system and available resources has been emphasised [6]. Indeed the methods and tools presented did not allow the evaluator to design his/her own evaluation process according to the surveillance context or to socio-economic constraints.

A further limitation of the existing approaches is the absence of a comprehensive list of attributes to be assessed, flexibility in the choice of attributes and guidance on how these should be selected. The updated CDC guidelines [26] did suggest that not all of attributes listed might be relevant and that they could be selected according to the context and the objectives of the evaluation. The descriptions of the developmental process provided in the reviewed literature were not sufficient to understand the process of attribute selection in the different approaches; if they were selected, e.g., due to their relative importance in the evaluation of surveillance systems, or due to the ease of assessment. Only one approach [28] provided a method for selecting relevant attributes according to the surveillance objectives. However, no guidance was provided in the document about how to perform this selection process.

There was limited guidance provided about the methods for assessment of attributes. Only one approach (clearly labelled as a tool) provided detailed methods for the assessment of attributes [27] but this allowed no flexibility in the selection of methods for the assessment of attributes. The selection of an appropriate assessment method could be complex and an evaluation approach should provide sufficient elements to help the evaluators’ choices. Indeed there is a need to review the advantages and limits of the current methods, as well as the required resources for their implementation (i.e. data required, technological requirement, and specific knowledge). The development of guidance for the selection of relevant attributes and the most appropriate methods to assess them would provide another degree of flexibility in the evaluation process itself.

In addition to this need for guidance on the selection and assessment of attributes there is also a need to include a comprehensive list of evaluation attributes that could be assessed. This review confirmed previous publication highlighting the need to consider economic attributes in the evaluation approaches (e.g. cost-effectiveness, cost-benefits) [3]. Economic aspects are a central issue in most decision processes and would allow for better selection and/or priorisation of efficient corrective actions. These elements could have an important role in defining the evaluation process as it would allow better targeting the evaluation considering the benefits for decision-makers who often need to make choices based on limited or diminishing resources [3]. There are needs regarding sociological attributes as well (e.g. acceptability, communication, non-monetary benefits), due to the fact that none of the evaluation approaches provided information on how to take into consideration stakeholders’ perceptions, needs and expectations. Moreover, evaluation should also take into consideration the needs and interests of the system’s stakeholders [34]. These aspects are essential to ensure the surveillance systems acceptability, sustainability and impact. It is important to understand stakeholders’ perceptions and expectations in order to ensure that the system is working properly and provides relevant information. As described in the paper by Auer and co-workers [34], acceptability can be considered as an underpinning attribute. Methods and tools to assess and evaluate these elements should be developed and included in the evaluation approaches.

None of the approaches provided gold standards which could guide the interpretation of the assessment results and target the corrective actions to be implemented. How to set the economic target would also need to be considered in the evaluation approaches in order to provide recommendations on how to balance performances versus costs, especially in situation where resources are scarce.

Other limitation of the existing approaches included the absence of recommendations about who should carry out the evaluation, which would help in setting up the evaluation, and of graphical representation of the outputs to assist with dissemination of the results. In addition a description of case study applications could assist end users in understanding how to implement the evaluation. Also, some transparency in the development process of the approaches would add to their usability by providing possibilities to see and evaluate possible conflicts of interest.

## Conclusion

Several organizations have developed evaluation approaches, targeting only partial aspects of the surveillance systems characteristic; and most of the available approaches provide general recommendations for evaluations.

This review highlighted the needs to develop a comprehensive approach for the evaluation of surveillance systems, based on the existing ones, and including guidance on the assessment of individual attributes. This approach would need to be *(i)* complete, i.e. to provide a full list of attributes not only covering the epidemiological aspects for the evaluation, but also the social and economic aspects; *(ii)* flexible and adaptable to the context (surveillance purpose and objective of the evaluation) and evaluations constraints (time, resources, available data, etc.); and *(iii)* operational, i.e. to provide a structured process for carrying out the evaluation which includes guidance on how to select appropriate attributes and the selection of practical methods and tools for their assessment.

## Endnote

^a^The overall aim of RISKSUR is to develop and validate conceptual and decision support frameworks and associated tools for designing efficient risk-based animal health surveillance systems http://www.fp7-risksur.eu/.

## Abbreviations

PH: Public health
AH: Animal health
EH: Environmental health
OH: One health
CCP: Critical control point
HACCP: Hazard analysis of critical control point
CDC: Centre for disease control and prevention
WHO: World health organisation
RESAPATH: Surveillance network for antimicrobial resistance in pathogenic bacteria from animal origin
RENESA: Evolution of mycoplasmosis and salmonellosis rates in poultry
FMD: Foot and Mouth Disease
OASIS: Outil d’analyse des systèmes de surveillance
SERVAL: SuRveillance EVALuation framework
